# Expression Profiles of miR-22-5p and miR-142-3p Indicate Hashimoto’s Disease and Are related to Thyroid Antibodies

**DOI:** 10.3390/genes13020171

**Published:** 2022-01-19

**Authors:** Olivia Trummer, Ines Foessl, Natascha Schweighofer, Edi Arifi, Christoph W. Haudum, Sharmaine Reintar, Stefan Pilz, Verena Theiler-Schwetz, Christian Trummer, Andreas Zirlik, Albrecht Schmidt, Caterina Colantonio, Ewald Kolesnik, Nicolas Verheyen, Thomas R. Pieber, Barbara Obermayer-Pietsch

**Affiliations:** 1 Division of Endocrinology and Diabetology, Department of Internal Medicine, Medical University of Graz, 8036 Graz, Austria; ines.foessl@medunigraz.at (I.F.); natascha.schweighofer@medunigraz.at (N.S.); edi.arifi@stud.medunigraz.at (E.A.); christoph.haudum@medunigraz.at (C.W.H.); sharmaine.reintar@medunigraz.at (S.R.); stefan.pilz@medunigraz.at (S.P.); verena.schwetz@medunigraz.at (V.T.-S.); christian.trummer@medunigraz.at (C.T.); thomas.pieber@medunigraz.at (T.R.P.); barbara.obermayer@medunigraz.at (B.O.-P.); 2 Center for Biomarker Research in Medicine, CBmed, 8010 Graz, Austria; 3 Division of Cardiology, Department of Cardiology, University Heart Center Graz, Medical University of Graz, 8036 Graz, Austria; andreas.zirlik@medunigraz.at (A.Z.); albrecht.schmidt@medunigraz.at (A.S.); caterina.colantonio@medunigraz.at (C.C.); ewald.kolesnik@medunigraz.at (E.K.); nicolas.verheyen@medunigraz.at (N.V.)

**Keywords:** miRNA, autoimmune thyroid disease, AITD, Hashimoto’s thyroiditis

## Abstract

Hashimoto’s thyroiditis (HT) is the most prevalent autoimmune disorder of the thyroid (AITD) and characterized by the presence of circulating autoantibodies evoked by a, to date, not fully understood dysregulation of the immune system. Autoreactive lymphocytes and inflammatory processes in the thyroid gland can impair or enhance thyroid hormone secretion. MicroRNAs (miRNAs) are small noncoding RNAs, which can play a pivotal role in immune functions and the development of autoimmunity. The aim of the present study was to evaluate whether the expression of 9 selected miRNAs related to immunological functions differ in patients with HT compared to healthy controls. MiRNA profiles were analysed using quantitative reverse transcription polymerase chain reaction (qRT-PCR) in 24 patients with HT and 17 healthy controls. Systemic expressions of miR-21-5p, miR-22-3p, miR-22-5p, miR-142-3p, miR-146a-5p, miR-301-3p and miR-451 were significantly upregulated in patients with HT (*p* ≤ 0.01) and were suitable to discriminate between HT and healthy controls in AUC analysis. Altered expressions of miR-22-5p and miR-142-3p were associated with higher levels of thyroid antibodies, suggesting their contribution to the pathogenesis of HT.

## 1. Introduction

Autoimmune thyroid diseases (AITDs) are the most common autoimmune diseases, affecting 2–5% of the population in high-income countries [1]. Hashimoto’s thyroiditis (HT), the most frequent AITD, is the leading cause of hypothyroidism in iodine-sufficient areas of the world. Although exact mechanisms of aetiology and pathogenesis of HT are not completely understood, a strong genetic susceptibility to the disease has been confirmed by studies carried out within families and twins [2]. As in other autoimmune disorders, humoral and cellular immune mechanisms are closely related and cross-linked in AITDs. Disturbed self-tolerance accompanied by an increased antigen presentation is a precondition for their manifestation, based also on the interaction of thyroid, antigen presenting and T cells. Secreted cytokines provoke predominantly a T-helper type 1 (Th1) as well as a Th17 response, which has been described [3]. Impaired thyroxin production and hypothyroidism as well as, more rarely, hyperthyroidism, are the consequences.

Early diagnosis and intervention may help to prevent the development of HT and abnormal thyroid function. The final diagnosis of HT depends on lymphocytic infiltration of the thyroid gland by fine-needle aspiration biopsy (FNAB) and further histopathological examination which is invasive and sometimes unfeasible [4]. Serum thyroid antibodies and ultrasonography are now used for diagnosis. At an early stage, HT is asymptomatic, easily leading to misdiagnosis [5]. Therefore, more biological markers need to be discovered to assist in early and accurate diagnosis of HT.

Micro RNAs (miRNAs) are small, noncoding, highly conserved ribonucleic acids (RNAs) that regulate gene expression by binding to messenger RNA (mRNA), thus modifying transcriptional processes. A single miRNA can regulate the expression of multiple genes and their encoded proteins [6]. In total, over 30% of human mRNAs are regulated by miRNAs [7]. Many miRNAs have been found to be important for the survival, development, differentiation, and function of T cells, B cells, dendritic cells, macrophages and other immune cell types [8,9]. Accordingly, differential miRNA expression profiles have been reported in autoimmunological disorders such as rheumatoid arthritis, systemic lupus erythematosus and psoriasis, [10,11,12,13,14] as well as in AITDs [15,16,17,18,19]. The aim of the study was to examine a panel of nine selected miRNAs to evaluate whether there is a difference in serum expressions of patients with HT and to investigate possible relations to thyroid antibodies. Candidate miRNAs for the present investigation have been selected according to their presence in serum as well as to previously described associations of humoral and/or cellular immune mechanisms involved in AITDs (Table 1).

## 2. Materials and Methods

### 2.1. Study Populations

Data of the present investigation were obtained from the BioPersMed cohort (“Biomarkers of Personalized Medicine”), an ongoing single-centre, prospective, observational study to evaluate novel biomarkers for the assessment of cardiovascular and common metabolic diseases and their related complications. This observational trial was initiated in the year 2010 and the study population consists of 1022 asymptomatic subjects without diagnosed cardiovascular disease (CVD) with at least one classical risk factor for CVD, such as family history of CVD, hypertension or dyslipidaemia. Extensive anthropometric and clinical data were carefully recorded, including comorbidities such as previously diagnosed HT. Patients presenting with severe illnesses independent of aetiology, or who were expected not to be able to complete study specific examinations, have been excluded from participation. Moreover, persons with serious co-morbidities or mental health problems have also been excluded. Written informed consent from each participant was obtained after the study approval by the institutional review board of the Medical University of Graz (EC Nr. 24-224 ex 11-12). The BioPersMed study is conducted in compliance with Good Clinical Practice Guidelines Procedures (GCP) and carried out according to the principles of the Declaration of Helsinki.

**Figure 1:**
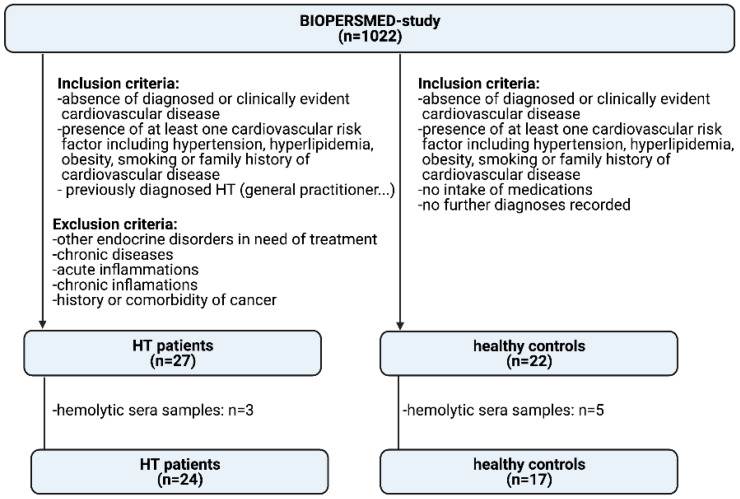
Study flow chart.

For the present observational investigation, we screened the BioPersMed cohort for previously diagnosed HT patients (*n* = 27) as well as age and sex matched participants suitable as healthy controls (*n* = 22). HT patients have been diagnosed based on the commonly used diagnostic tools such as clinical manifestations, ultrasound and measurement of thyroid stimulating hormone (TSH), free triiodothyronine (fT3), free thyroxine (fT4), thyroglobulin autoantibody (TgAb) and thyroid peroxidase autoantibody (TPOAb) by their general practitioner or any other medical facilities. Exclusion criteria for HT patients were comorbidities such as acute (e.g., pancreatitis) or chronic inflammations (e.g., rheumatoid arthritis, polymyalgia, diabetes mellitus), endocrine disturbances in need of treatment (other than HT), history of myocardial infarction as well as history of cancers (e.g., bladder cancer, acoustic neuroma). Participants in the healthy control group showed at least one classical risk factor for CVD, but no serious comorbidities after a clinical validation by an experienced clinician. Serum samples were excluded if haemolysis was visually detected. We therefore excluded 3 samples of the HT group and 5 samples of the control group. In total, we investigated 24 HT patients compared to 17 healthy controls. A study flow chart is given in Figure 1.

### 2.2. Patient Visit

Anthropometric data were measured in all participants. Baseline blood samples for laboratory analyses were collected between 7.00 and 9.00 a.m. after an overnight fast. Biobanking of blood samples was performed by freezing and storing the samples at −80 °C until analysis. To evaluate thyroid function and common autoantibodies, serum levels of TSH, fT3, fT4, TPOAb and TgAb were determined by luminescence immunoassay (Siemens, Erlangen, Germany) with intra- and inter-assay coefficients of variation (CV) of: TSH, 5.0% and 6.0%; FT3, 2.4% and 2.9%; FT4, 2.2% and 2.3%; TPO Ab, 5.2% and 6.1%, as well as Tg Ab, 5.0% and 4.6%, respectively. Body mass index (BMI) was calculated as body weight in kilograms (kg) divided by height in meters squared (m^2^).

### 2.3. Selection of miRNAs

Based on previous studies [15,16], we selected 9 miRNAs that have been related to relevant immunological functions as candidates for the present investigation. These miRNAs are listed in Table 1.

### 2.4. miRNA Isolation and qPCR

MiRNA was isolated using the miRNeasy Serum/Plasma Advanced Kit (Qiagen, Hilden, Germany) according to the manufacturer’s instructions. RNA was eluted from the columns by addition of 20 μL RNase-free water, followed by centrifugation. The isolated miRNAs were short-term stored at −80 °C. Complementary DNA (cDNA) was generated using miRCURY LNA RT synthesis kit (Qiagen, Hilden, Germany), and subsequent quantitative real-time PCR (qPCR) was performed in duplicates using miRCURY LNA SYBR Green PCR Kit and specific miRCURY LNA miRNA PCR Assays (both from Qiagen, Hilden, Germany) with the CFX384 Touch Real-Time PCR Detection System (Bio-Rad, Hercules, CA, USA). Exogenous oligonucleotides have been added as spike-in controls (UniSp2, UniSp4, UniSp5, UniSp6 and cel-miR-39-3p) and were used to estimate the efficiency of RNA extraction, reverse transcription reaction and qPCR amplification (RNA Spike in Kit for RT, Qiagen, Hilden, Germany). All qPCRs were performed with interplate calibration, a maximum of 40 cycles were performed in duplicates and the average of cycle threshold (Ct) values were calculated. Only those miRNAs with a Ct < 37 were considered for further analysis. The relative expression levels of all investigated miRNAs were calculated as fold change [20]. For that, average Ct values have been normalized to spike-in controls to calculate ΔCt values. Fold change was calculated as 2^−ΔΔCt^ where ΔΔCt was ΔCt of HT patients minus ΔCt of controls. Quantitative qPCR data are reported as mean ± standard deviation (SD).

### 2.5. Functional Annotation of miRNAs

MiRWalk was used to identify potential target genes of differentially expressed miRNAs [21]. Matched binding sites have been evaluated in genes reportedly involved in the development of AITDs [22].

### 2.6. Statistical Analysis

Statistical analysis was performed using SPSS statistics version 25.0 (IBM SPSS Statistics GmbH, Ehringen, Germany). Patient characteristics and biomarker results are reported as mean ± SD unless otherwise stated. Distribution of data was analysed by descriptive statistics and Kolmogorov–Smirnov test, as well as by evaluation of quantile-quantile plots. Normally distributed quantitative data were compared using unpaired Student’s *t*-test and unequally distributed data by applying Kruskal–Wallis tests for non-parametric samples. Changes of miRNA in the HT group are displayed as relative change compared to miRNA levels of healthy controls as reference. The diagnostic value for discriminating between HT patients and the control group was assessed by calculating the area under the curve (AUC). Receiver-operator characteristic (ROC) curves were generated by plotting sensitivity vs. (1-specificity). A *p*-value of ≤0.05 was considered as statistically significant. Adjustment for multiple testing has been performed by Bonferroni correction.

## 3. Results

### 3.1. General Results

We included a total of 41 participants, 33 women (81%), and 8 men (19%) in our analysis. Of these, 24 subjects (59%) were patients with previously diagnosed HT (22 women (92%) and 2 men (8%)). 17 subjects (41%) were classified as healthy (11 women (65%) and 6 men (35%)) (Figure 1).

In the HT patients group, 13% (*n* = 3) showed TgAb > 60 U/mL and 54% (*n* = 13) showed TPOAb > 60 U/mL. In total, 4 patients had both TgAb as well as TPOAb > 60 U/mL, respectively.

Of the HT patients, 29% (*n* = 7) were treated with levothyroxine. Control group participants did not take any recorded medication. Demographic data of the study population are given in Table 2.

### 3.2. miRNA Expression Is Altered in Patients with HT

**Figure 2:**
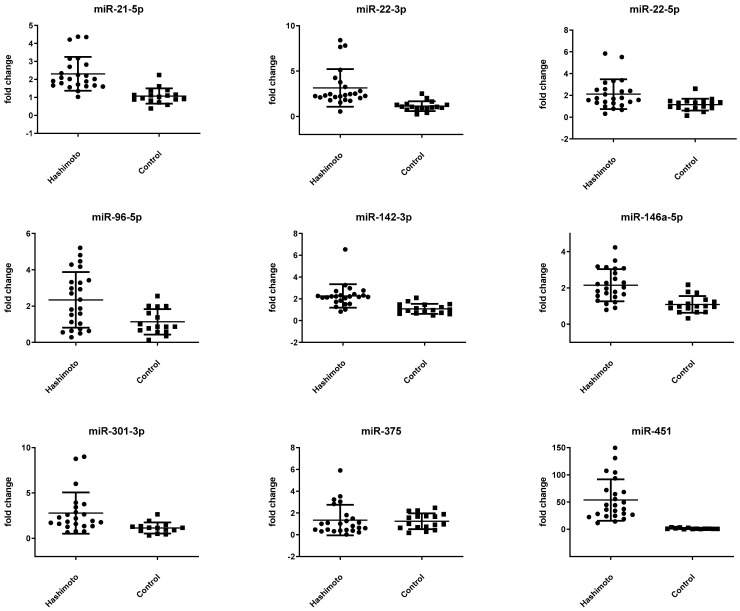
Expression of 9 miRNAs in serum of samples of HT patients and healthy controls. Data are displayed as scatter plots, where each dot represents the fold change as 2^−^^ΔΔ^^ct^-value of one study sample. Significance was tested by unpaired Student’s *t*-test.

Systemic expression of miR-21-5p, miR-22-3p, miR-22-5p, miR-96-5p, miR-142-3p, miR-146a-5p, miR-301a-5p, and miR-451 was significantly upregulated in patients with HT. Associations of miR-21-5p, miR-22-3p, miR-142-3p, miR-146a-5p, miR-301-3p as well as miRNA-451 remained stable after Bonferroni correction. In contrast, miRNA-22-5p and miRNA-96-5p lost the level of significance after adjustment for multiple testing. Out of the nine selected miRNAs, miR-375 was the only candidate that was not upregulated in serum of HT patients. (Table 3). ΔCt values per group were normally distributed. Respective scatter plots are given in Figure 2. An annotation in miRWalk provided information on binding sites of the potential miRNAs.

### 3.3. miR-22-5p and miR-142-3p Are Altered in HT Patients with Higher Levels of Thyroid Antibodies

Subgroup analyses within HT patients showed significantly higher miRNA expression for miR-22-5p in HT patients with higher thyroid antibody levels (TgAb and/or TPOAb > 60 U/mL, *n* = 13), 5.97 ± 0.74, as compared to HT patients with lower thyroid antibody levels (TgAb and/or TPOAb < 60 U/mL, *n* = 11) 6.95 ± 0.90; *p* = 0.008.

**Figure 3:**
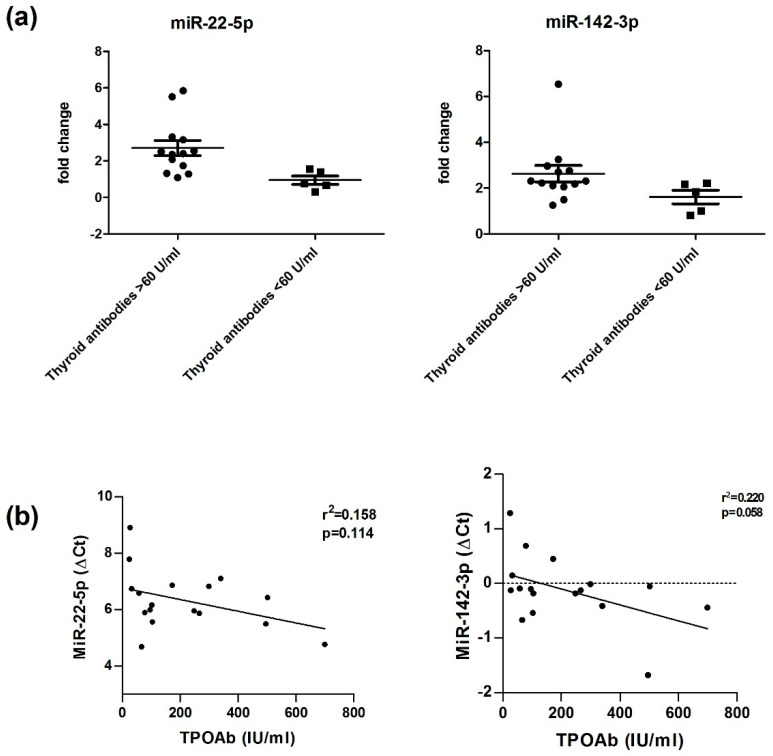
(**a**) Altered Expressions of miR-22-5p and miR-142-3p in serum of HT patients with higher levels of thyroid antibodies compared to HT patients with thyroid antibody levels <60 U/mL. Data are displayed as scatter plots, where each dot represents the ΔCt value of one HT patient. Significance was tested by unpaired Student’s *t*-test. (**b**)Thyroid Antibodies (TPOAb) did not correlate with higher expressions of miR-22-5p and miR-142-3p (ΔCt values). TgAb, thyroglobulin autoantibody; TPOAb, thyroid peroxidase autoantibody; ΔCt, delta Cycle threshold.

MiR-142-3p was also found to be significantly different (*p* = 0.05) in HT patients with higher levels of thyroid antibodies −0.25 ± 0.57 as compared to HT patients with thyroid antibody levels <60 U/mL, 0.21 ± 0.51. In our regression analysis miR-22-5p and miR-142-3p expressions did not correlate with TPOAb levels (Figure 3). 

### 3.4. Potential Binding Site Targets

**Figure 4:**
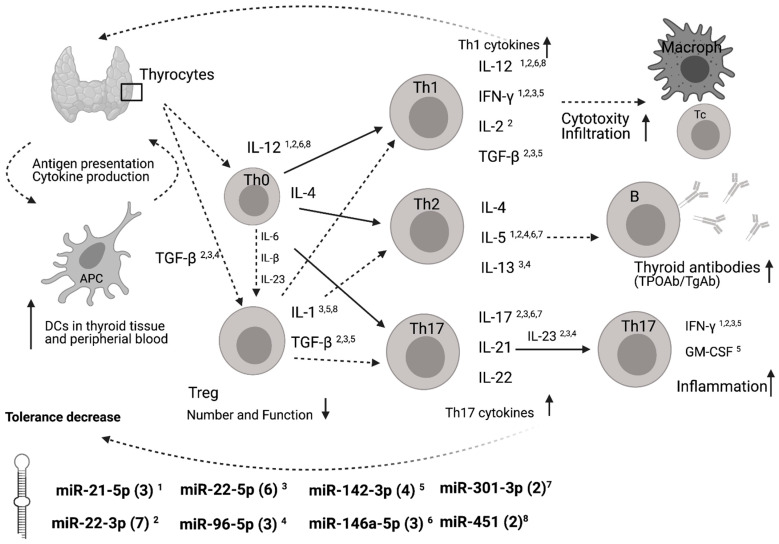
Summary of the main mechanisms related to autoimmunity of HT and potential interaction sites with differentially expressed miRNAs. Schematic representation of T cells differentiating into specific T cell subsets depending on the cytokines to which they are exposed and their main effects. MiRNA binding site predictions have been annotated by miRWalk database. Number of predicted binding sites are shown in brackets for each miRNA. Predicted binding sites of genes of HT immune-related molecules and/or their receptors are marked by superscript numbers. Adapted from [22]. APC, antigen presenting cell; Th, T helper cell; Macroph, macrophage; DC, dentritic cell; Treg, T-regulatory cells; TPOAb; peroxidase autoantibody; TgAb, thyroglobulin autoantibody, IL, interleukin; IFN-γ, interferon-γ; TGF-β, transforming growth factor β; GM-CSF, granulocyte-macrophage colony-stimulating factor; miR, miRNA.

A functional annotation of these differentially expressed miRNAs revealed potential binding sites in genes of important immune mediators such as interleukins (IL), interferons (IFN), transforming growth factors (TGF), and granulocyte-macrophage colony-stimulating factor (GM-CSF). A scheme on how these miRNAs may interact in the development of AITD is given in Figure 4. Higher numbers of predicted miRNA binding sites were determined for miR-22-5p (*n* = 6) and for miR-142-3p (*n* = 4) compared to the mean number of binding sites for all miRNAs of 3.3 ± 2.0 (SD). MiR-21-5p showed potential interactions with IL-5, IFN-γ and IL-12. MiR-22-3p potentially interacts with IL-2, IL-5, IL-12, IL-17, IL-23, IFN- γ and TGF-β. MiR-22-5p potentially interacts with IL-1, IL-12, IL-13, IL-17 and IL-23 and IFN-γ. MiR-96-5p potentially interacts with IL-5, IL-13 and IL-23. MiR-142-3p potentially interacts with IL-1, TGFβ, IFN-γ and GM-CSF. MiR-146a-5p potentially interacts with IL-12, IL-5 and IL-17. MiR-301-3p potentially interacts with IL-7 and IL-17. MiR-451 potentially interacts with IL-1 and IL-12. 

### 3.5. miRNAs as Discriminators for HT Status in ROC Analysis

**Figure 5:**
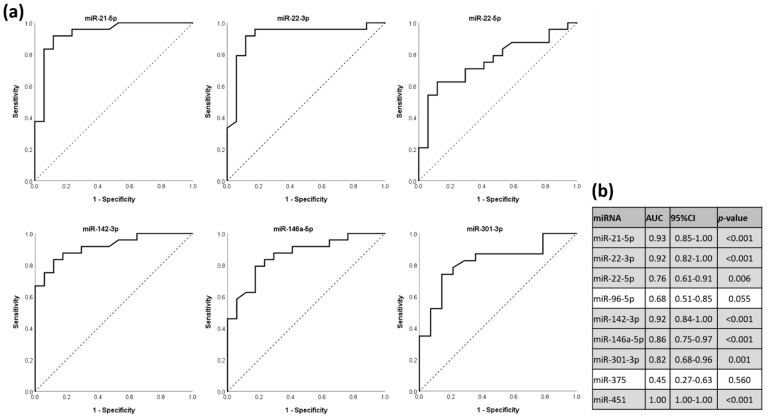
The potential of differentially expressed miRNAs to discriminate between HT status and healthy controls, shown in ROC curves. (**a**) ROC curves of selected differently expressed miRNAs. (**b**) Calculated AUC values, 95% CI and *p*-values of each investigated miRNA. ROC, receiver-operating characteristic; DE, differentially expressed; AUC, area under the curve; CI, confidence interval.

To evaluate the discriminatory potential of the differentially expressed miRNAs, we performed receiver-operating characteristic (ROC) analysis and calculated area under the curve (AUC) values. With the exception of miR-96-5p, differentially expressed miRNAs are “fair” (miRNA 22-5p: AUC = 0.76; 95% CI, 0.61–0.91; *p* = 0.006), “good” (miR-301-3p: AUC = 0.82; 95% CI, 0.68–0.96; *p* = 0.001 and miR-146a-5p: AUC = 0.86; 95% CI, 0.75–0.97; *p* < 0.001) or “excellent” (miR-21-5p: AUC = 0.99; 95% CI, 0.85–1; *p* < 0.001; miR-22-3p: AUC = 0.92; 95% CI, 0.82–1.00; *p* < 0.001; miR-142-3p: AUC = 0.92; 95% CI, 0.84–1.00; *p* < 0.001 and miR-451: AUC = 1.00; 95% CI, 1.00–1.00; *p* < 0.001) predictors [23] (Figure 5).

## 4. Discussion

In the present observational study, eight out of nine observed miRNAs were differentially expressed in the serum of HT patients compared to healthy controls. In a subgroup analysis, HT patients with thyroid antibodies (TPOAb and/or TgAb) showed significantly higher expression levels for miR-22-5p and miR-142-3p but not for the other 7 miRNAs (Figure 3). To evaluate the accuracy of these differentially expressed miRNAs in predicting HT, we conducted a ROC analysis. AUC of these miRNAs were at least >0.76, indicating a “fair” test with enough balance between sensitivity and specificity for discriminating accurately between HT patients and healthy controls [24], (Figure 5).

The general tendency to overexpression detectable for all investigated miRNAs might be due to either active secretion as a consequence of increased inflammation in HT patients or derivation from autoimmune-related cell death. Independent from their cells of origin, miRNAs can function as endocrine signallers and are taken up by target cells [25].

We detected an increased expression of miR-22-5p and miRNA-142-3p, both associated with higher levels of serum antibodies (TgAb and/or TPOAb > 60 U/mL) in patients with HT. This corresponds with data of our ROC analysis. Both miRNAs are suitable to discriminate between HT patients and healthy controls. We could not confirm the association between antibody level group and altered expression by correlation analysis. Thyroid antibodies did not correlate with expression levels of miR-22-5p and miR-142-3p (Figure 3). Furthermore, and this counts for all of our analyses, we cannot estimate how the investigated miRNAs vary in their expressions before, during or after development of HT since this study was observational after development of HT.

Our results are in context with previously published data. Zhu et al. reported positive associations of TgAb levels and miR-142-5p but not for miR-142-3p [26], which is in line with our thyroid antibody analysis. Data of ROC curves are corresponding with ROC curves of Martínez-Hernández et al. with the exception of miR-96-5p. According to their analysis this miRNA is an “excellent” predictor (AUC = 0.91, 95% CI, 0.84–0.98) whereas our data failed the level of significance barely (*p* = 0.055) [24].

Our data of overexpression profiles in HT are in accordance with Martínez-Hernández et al. [15] as well as Yamada et al. [16], who both studied miRNA expressions in patients with AITD. The only non-concordant exception is miR-375, which was not differentially expressed in our cohort in contrast to Yamada’s study. That in turn is in line with Zhao et al. who showed upregulated plasma levels of miR-375 and miR-451 in a four times larger study cohort than Yamada [5]. We are aware that caution must be taken when comparing miRNA data generated from different types of biofluids [27], since serum and plasma differ in their content of miRNA derived from different blood cells [28,29]. One of the potential reasons for the partly conflicting results of studies on miRNA expression in HT patients may be the interethnic expression differences between Asian (Yamada et al., Zhu et al.) and Caucasian (present study) cohorts [30]. In both investigations, serum miRNA profiling was performed by reverse transcription qPCR, the gold standard for sensitive and specific quantification of miRNAs in cell free biofluids. However, as frequently encountered, the quality of results varies in general strongly with the preanalytical steps such as blood drawing and serum/plasma preparation. The very low amounts of miRNAs, potentially high levels of inhibitors, biological variances of the individuals themselves (diet, exercise, age) as well as normalization strategies contribute to the variance in the results of different miRNA studies [31,32,33].

As confirmed by our in silico analysis, several T cell differentiation cytokines related to autoimmunity are potential targets of our overexpressed miRNA patterns (Figure 4). 

MiR-22-3p binds 7 autoimmune-related cytokines, miR-22-5p has 6 binding partners and miR-142-3p has 4 binding partners. This may suggest a contribution of these miRNAs to differentiation of T cells into specific T cell subsets. MiR-22-5p regulates mainly cytokines involved in the differentiation of Th17 cells, promoting therefore the inflammatory response. Our focus was on miRNA targets related to autoimmunity of HT. We are aware that such stringent criteria potentially excluded other regulatory interactions that might also play a role in the development of HT.

In our investigation miR-22-3p is associated with HT but not to serum thyroid antibodies. MiR-22-3p mainly targets genes of Th1 cytokines (IL-12, IFN-γ, IL-2 and TGF-β) but is binding partner of fewer TH2 cytokine genes (IL5) (Figure 4). This might suggest that miR-22-3p rather regulates the autoimmune related cytotoxicity and infiltration than changes in the development of thyroid antibodies. These theoretical assumptions are based on our in silico analysis. Whether cytokine levels are affected by the changed miRNA profile remains to be elucidated. This study was focussing on the biomarker aspect of the miRNA pattern. Nevertheless, our data showed serum overexpression of miR-22-5p and miR-142-3p related to the occurrence of thyroid antibodies.

Some further limitations of the study should be taken into account. There is still a debate on how and if normalization should be performed on qPCR results of serum miRNAs [34]. We decided to use exogenous controls (spike-ins) for normalization, excluding a potential bias by normalization on endogenous reference genes [33]. We cannot rule out a certain selection bias by choosing HT patients based on their previous medical history and not their prospective enrolment into the study. Further, cytokine levels were not determined in the BioPersMed cohort at the time of the patients’ visits.

It should be kept in mind that published data, including the present study, lack long-term outcome data regarding disease activity and prognostic expectations. In this study, we present 2 miRNA candidates associated with higher occurrence of thyroid antibodies that possibly could be suitable to allow assumptions on whether HT patients are likely to develop higher titers of thyroid antibodies (TPOAb and or TgAb < 60 U/mL).

In conclusion, miRNA profiles of miR-21-5p, miR-22,3p, miR-22-5p, miR-142-3p, miR-146a-5p, miR-301-3p and miR-451 are upregulated in HT patients and suitable to discriminate between HT and healthy controls. Additionally, altered expressions of miR-22-5p and miR-142-3p are associated with higher levels of thyroid antibodies, suggesting important roles in the pathogenesis of HT.

## Figures and Tables

**Table 1:** MiRNAs, mature sequence and source of reference of each selected miRNA.

micro RNAs	Sequence	Reference
hsa-miR-21-5p	5′UAGCUUAUCAGACUGAUGUUGA	[15]
hsa-miR-22-3p	5′AAGCUGCCAGUUGAAGAACUGU	[16]
hsa-miR-22-5p	5′AGUUCUUCAGUGGCAAGCUUUA	[16]
hsa-miR-96-5p	5′UUUGGCACUAGCACAUUUUUGCU	[15]
hsa-miR-142-3p	5′UGUAGUGUUUCCUACUUUAUGGA	[15]
hsa-miR-146a-5p	5′UGAGAACUGAAUUCCAUGGGUU	[15]
hsa-miR-301-3p	5′CAGUGCAAUAGUAUUGUCAAAGC	[15]
hsa-miR-375	5′UUUGUUCGUUCGGCUCGCGUGA	[16]
hsa-miR-451	5′AAACCGUUACCAUUACUGAGUU	[16]

hsa, homo sapiens; miRNA, micro RNA.

**Table 2:** Demographic data of patients with Hashimoto’s thyroiditis and healthy subjects. Frequency data are presented as number, (percentage), continuous data as mean ± standard deviation. HT, Hashimoto’s thyroiditis; n, number; BMI, body mass index; FT3, free triiodothyronine; FT4, free thyroxine; TSH, Thyroid stimulating hormone; TPOAb, thyroid peroxidase autoantibody; TgAb, thyroglobulin autoantibody. Normal ranges of fT3, 3.0–6.3 pmol/L; fT4, 9.5–24 pmol/L; TSH, 0.10–4.0 µU/mL; TgAb; 0–60 IU/mL; TPOAb, 0–60 IU/mL.

	Patients with HT	Healthy Subjects
*n*	24	17
Sex female (*n*)	22 (91.7%)	11 (64.7%)
Age (yr)	57.9 ± 7.4	61.1 ± 5.8
BMI (kg/m^2^)	25.2 ± 4.2	23.7 ± 2.34
fT3 (pmol/L)	4.4 ± 0.5	4.8 ± 0.4
fT4 (pmol/L)	15.9 ± 2.3	16.3 ± 2.0
TSH (µU/mL)	1.60 ± 0.87	1.83 ± 0.73
TgAb (IU/mL)	213.8 ± 365.6	
TPOAb (IU/mL)	212.2 ± 199.1	
Levothyroxine treatment	7 (29%)	

**Table 3:** MiRNA ΔCt values according to the selected miRNAs of patients with Hashimoto’s thyroiditis and healthy subjects. Data are shown as mean ± standard deviation.

hsa-miRNA	Patients with HT (*n* = 24)	Healthy Subjects (*n* = 17)	*p*-Value
miR-21-5p	−0.43 ± 0.54	0.68 ± 0.58	<0.001 *
miR-22-3p	1.58 ± 0.85	2.98 ± 0.80	<0.001 *
miR-22-5p	6.42 ± 0.95	7.22 ± 0.91	0.010
miR-96-5p	8.40 ± 1.21	9.22 ± 1.22	0.040
miR-142-3p	−0.84 ± 0.58	1.03 ± 0.60	<0.001 *
miR-146a-5p	2.71 ± 0.63	3.69 ± 0.65	<0.001 *
miR-301-3p	6.09 ± 1.01	7.21 ± 0.82	0.001 *
miR-375	8.52 ± 1.67	8.21 ± 1.10	0.503
miR-451	−2.60 ± 1.00	2.82 ± 1.35	<0.001 *

* indicates significant *p*-values after Bonferroni correction.
